# Effect of Annealing Process on the Properties of Ni(55%)Cr(40%)Si(5%) Thin-Film Resistors

**DOI:** 10.3390/ma8105338

**Published:** 2015-10-02

**Authors:** Huan-Yi Cheng, Ying-Chung Chen, Pei-Jou Li, Cheng-Fu Yang, Hong-Hsin Huang

**Affiliations:** 1 Department of Electrical Engineering, National Sun Yat-sen University, Kaohsiung 804, Taiwan; eveflora818@gmail.com (H.-Y.C.); ycc@mail.ee.nsysu.edu.tw (Y.-C.C.); sunnyrain516@hotmail.com (P.-J.L.); 2 Department of Chemical and Materials Engineering, National University of Kaohsiung, No. 700 Kaohsiung University Road, Nan-Tzu District, Kaohsiung 811, Taiwan; 3 Department of Electrical Engineering, Cheng Shiu University, Kaohsiung 833, Taiwan; k0511@gcloud.csu.edu.tw

**Keywords:** Ni(55%)Cr(40%)Si(5%), thin-film resistor, deposition time, rapid thermal annealing

## Abstract

Resistors in integrated circuits (ICs) are implemented using diffused methods fabricated in the base and emitter regions of bipolar transistor or in source/drain regions of CMOS. Deposition of thin films on the wafer surface is another choice to fabricate the thin-film resistors in ICs’ applications. In this study, Ni(55%)Cr(40%)Si(5%) (abbreviated as NiCrSi) in wt % was used as the target and the sputtering method was used to deposit the thin-film resistors on Al_2_O_3_ substrates. NiCrSi thin-film resistors with different thicknesses of 30.8 nm~334.7 nm were obtained by controlling deposition time. After deposition, the thin-film resistors were annealed at 400 °C under different durations in N_2_ atmosphere using the rapid thermal annealing (RTA) process. The sheet resistance of NiCrSi thin-film resistors was measured using the four-point-probe method from 25 °C to 125 °C, then the temperature coefficient of resistance could be obtained. We aim to show that resistivity of NiCrSi thin-film resistors decreased with increasing deposition time (thickness) and the annealing process had apparent effect on the sheet resistance and temperature coefficient of resistance. We also aim to show that the annealed NiCrSi thin-film resistors had a low temperature coefficient of resistance (TCR) between 0 ppm/°C and +50 ppm/°C.

## 1. Introduction

BiCMOS can provide superior performance for the applications in different circuits. Thus, the analog functions in BiCMOS require passive circuit components having small temperature coefficients, and the process sequence must be able to accommodate their fabrication. Resistors in integrated circuits (ICs) are implemented using diffused methods fabricated in the base and emitter regions of bipolar transistor or in source/drain regions of CMOS [1,2]. To deposit thin films on the wafer surface is another way to fabricate the passive resistors. Thin-film resistors are used extensively in electronic circuits due to their high accuracy and excellent long term stability. So far, there are various specific materials currently used as thin-film resistors in ICs’ applications, such as Cr–Si–Ta–Al [3], Ti–Si–V–N [4], and CuAlMo [5], respectively. One of the most widely used thin-film resistors is nickel–hromium (NiCr) which has a low temperature coefficient of resistance (abbreviated as TCR) between −50 ppm/°C and +50 ppm/°C [6,7] and has a wide sheet resistance range of 10 to 500 Ω/square [8]. Ni–Cr thin-film-based resistors are extensively used as discrete loads or potentiometers in hybrid circuits. Excellent wear and corrosion resistance makes Ni–Cr thin films an attractive material for fusible links in programmable read only memories [6]. Recently, there has been an increase in demand for thin-film resistors with lower value of 0.1~10 Ω, especially in portable electronic devices for the purpose of saving battery power [9]. This requirement is difficult to meet with the properties of NiCr-based thin-film resistors, because as the thickness of NiCr-based thin-film resistors increases, the cost of deposition process increases and problems at the subsequent laser trimming stage will occur, because they become difficult to ablate.

There are different materials currently used as thin-film resistors in IC applications and Cr–Si thin films are very interesting materials. The Cr–Si thin films can be deposited as the thin-film-resistors because of existing certain advantages, including high sheet resistance, low TCR, high thermal stability, good long-term reliability, and chemical stability, respectively [10]. Dong *et al.* previous investigation proved that Cr–Si (Cr:Si = 1:3) thin films doping with 3–6 at % Ni had the resistivity of 1.31–1.49 times higher than that of Cr–Si thin films without Ni [11,12]. They also found that the TCR of thin films could be adjusted to close zero by using annealing process. However, the resistivity of Cr–Si–Ni films was decreased when Ni content was higher than 6 at %. The results suggested that a moderate Ni addition, for example, equal and higher than 40 wt % of Ni in Cr films [7] or equal and higher than 6 at % of Ni in Cr–Si films was favorable to the electrical stability [11]. In this study, Ni(55%)Cr(40%)Si(5%) (abbreviated as NiCrSi) in wt % was used as the target because the NiCrSi thin films had the merit of long-term stability [13] and the sputtering method was used to deposit the thin-film resistors on glass substrates. After deposition, the thin-film resistors were also annealed at 400 °C under different duration using the rapid thermal annealing (RTA) process. The sheet resistance of thin-film resistors was measured using the four-point-probe method. We would show that resistivity of NiCrSi thin-film resistors decreased with increasing deposition time and the annealing process had no apparent effect on the value of sheet resistance and the TCR.

## 2. Experimental Section

**Figure 1:**
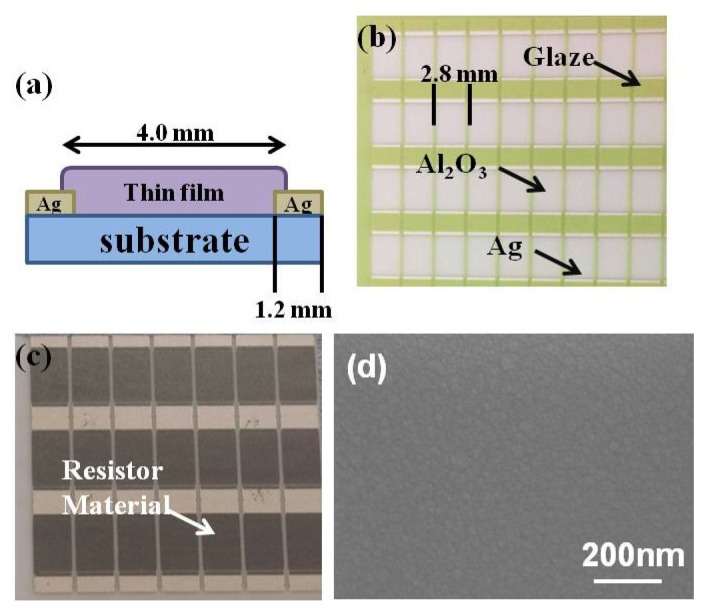
The structure of NiCrSi thin-film resistors (**a**) Side view; (**b**) top view before the protection glaze (green color) films were removed; and (**c**) top view, the protection glaze films were removed after the resistor material was deposited; (**d**) Surface morphology of deposited NiCrSi thin-film resistors.

The structure of deposited NiCrSi-based thin-films using for the measurements of physical and electrical properties was shown in Figure 1. Figure 1a shows the side view of the structure. The green glass paste on Al_2_O_3_ ceramic shown in Figure 1b was used to protect the substrate, and it could be removed during the NiCrSi thin-film resistors’ annealing process and following ultrasonic clean in deionized water. The length between two electrodes (or called the length of thin-film resistors) was 4.0 mm, the widths of thin-film resistors and electrodes were 2.8 mm, and the length of electrodes was 1.2 mm, respectively, as Figure 1a,b show. Commercial composition material Ni(55%)Cr(40%)Si(5%) (abbreviated as NiCrSi) in wt % was used as the target and the sputtering method was used to deposit NiCrSi thin films on Al_2_O_3_ substrates. The deposition parameters were power of 150 W at 5 mTorr and at room temperature (25 °C), and the deposition time was changed from 30 min to 150 min, respectively. After deposition, the deposited NiCrSi thin films were annealed by using the rapid thermal annealing (RTA) at 400 °C in N_2_ under different durations of 30 s~10 min. After NiCrSi-based thin-films were annealed, the glaze layer was removed by using ultrasonic method in deionized water. The surface morphology of deposited NiCrSi thin-film resistors was shown in Figure 1c. The crystalline structures of deposited and annealed NiCrSi-based thin-films were determined by means of X-ray diffraction (XRD) (Cu–Kα, Bruker D8, Billerica, MA, USA). The thickness measurement of deposited and annealed Ni–Cr–Si-based thin-films was obtained by using α-step (Kosaka ET 4000, Tokyo, Japan).

The surface morphology of NiCrSi thin-film resistors was observed by field emission scanning electron microscopy (FESEM) (JEOL JSM-6700, Tokyo, Japan). As Figure 1d shows, only nano-crystalline grains were observed and surface morphology was almost unchanged as the different deposition time and the annealing process were used. The resistance was measured by using the four-point-probe method and the resistivity was calculated by the measured resistance and the thickness of Ni–Cr–Si-based thin films. Resistance values for materials at any temperature other than the standard temperature (usually specified at 20 °C) on the specific resistance table can be determined through the following formula:

R = R_ref_ [1 + α (T − T_ref_)] (1)

where R is the material resistance at temperature T, R_ref_ is the material resistance at temperature T_ref_, usually 20 °C or 0 °C, α is temperature coefficient of resistance (TCR) for the material symbolizing the resistance change factor per degree of temperature change, T is the material temperature in degrees Celcius, and T_ref_ is the reference temperature that α is specified at for the material, respectively. In this study, the measured temperatures were 25 °C, 50 °C, 75 °C, 100 °C, and 125 °C, respectively, the resistivity measured at the two temperatures were used to find the temperature coefficient of resistance of NiCrSi thin-film resistors.

## 3. Results and Discussion

**Figure 2:**
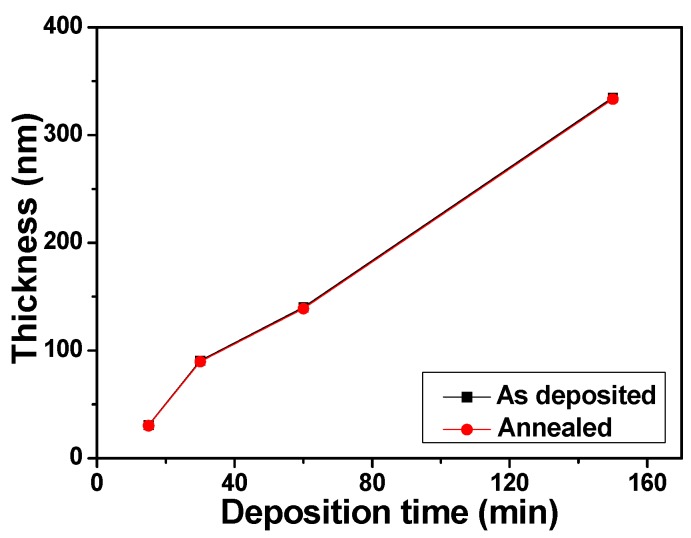
Thickness of as-deposited and annealed NiCrSi thin-film resistors.

The effect of deposition time on the thickness of as-deposited and annealed NiCrSi thin films was investigated, and the results are shown in Figure 2. As the deposition temperature was 25 °C, the thicknesses of the 15 min-, 30 min-, 60 min-, and 150 min-deposited NiCrSi thin films measured by using Ellipsometer and the thickness were around 30.8 nm, 90.7 nm, 140.1 nm, and 334.7 nm, respectively. As the deposition time increased, the increase in thickness of NiCrSi thin films is expectable. However, as NiCrSi thin films were annealed at 400 °C in N_2_ atmosphere, the thicknesses of the 15 min-, 30 min-, 60 min-, and 150 min-deposited NiCrSi thin films decreased slightly.

**Figure 3:**
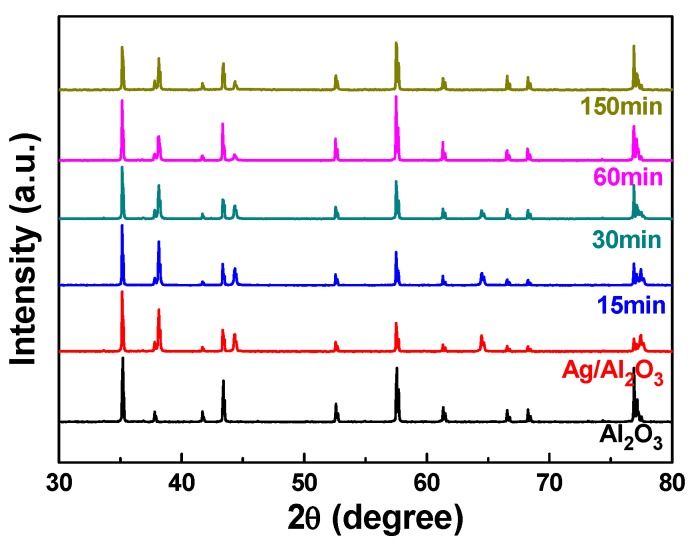
X-ray diffraction (XRD) patterns of deposited NiCrSi thin-film resistors.

XRD was used to investigate the structural properties of NiCrSi thin films deposition at room temperature in a pure Ar atmosphere. Figure 3 shows the XRD patterns of Al_2_O_3_ substrate, Al_2_O_3_ substrate with Ag paste. Figure 3 also shows the XRD patterns of the 15 min-, 30 min-, 60 min-, and 150 min-deposited NiCrSi thin films with the different thicknesses. As Figure 3 shows, the XRD patterns of all deposited NiCrSi thin films revealed an amorphous structure and no apparent crystalline phases were observed. Only the Ag and Al_2_O_3_ phases were observed in Figure 3. Those results suggest that the thickness (or deposition time) will not affect the crystalline structure of deposited NiCrSi thin films.

**Figure 4:**
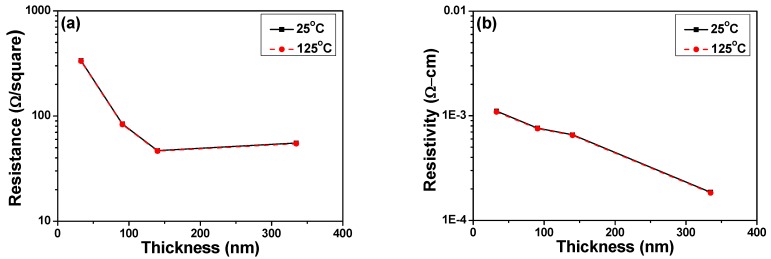
Effect of thickness on the variations of (**a**) resistance and (**b**) resistivity for NiCrSi thin-film resistors measured at 25 °C and 125 °C.

Figure 4 shows the effect of thickness on the variations of resistance and resistivity for NiCrSi thin-film resistors measured at 25 °C and 125 °C. The NiCrSi thin-film resistors’ resistance was recorded by the four-point measurement and the resistivity was derived from the resistance using a measurement of film thickness. As Figure 4 shows, as the measured temperatures were increased from 25 °C to 125 °C (only measured at 25 °C to 125 °C were shown); however, the resistance of NiCrSi thin-film resistors increased slightly and they had similar results. Figure 4 also shows that the thinner NiCrSi thin-film resistors showed higher resistivity and the resistivity reached a saturation value as the thickness was equal and more than 140.1 nm. If we suppose the thickness of NiCrSi thin-film resistors was independent of measured temperature, the resistivity of the 25 °C- and 125 °C-measured NiCrSi thin-film resistors had almost the same values and the variations in resistivity were not apparently observed. Figure 4 shows that the resistivity linearly decreased with increasing NiCrSi thin-films’ thickness. In the free-electron model of a metallic thin film with hard-wall boundary conditions, the discretization of energy levels makes it impossible to treat both the Fermi energy and the electron density as independent of the thickness [14].

Nevertheless, many scattering effects are believed to affect the resistivity of NiCrSi thin-film resistors, including surface scattering effect, grain boundaries scattering effect, uneven or rough surfaces scattering effect, and impurities scattering effect, respectively [15]. Surface scattering effect is dependent on the thickness of thin-film resistors and other effects are dependent on the procedures and conditions used to fabricate the thin films, and thus, it is very difficult to quantify each of these effects without measurement [15]. From the Lacy’s propose, the electrical resistivity as a function of film thickness can be expressed as:

(2) $$\rho =\;\frac{{\text{ρ}}_{\text{o}}}{k\;\left[\;1-\ln k\right]}\;$$

(3) $$\text{and }k=\;\frac{t/2}{l}$$

where *k* is constant and 0 < *k* ≤ 1, ρ is resistivity of thin film resistors, ρ_o_ is the bulk resistivity of the material, *l* is the average traveling distance of electrons an, *t* is the thickness of thin film conductors and it is assumed to have smooth or even surfaces.

**Figure 5:**
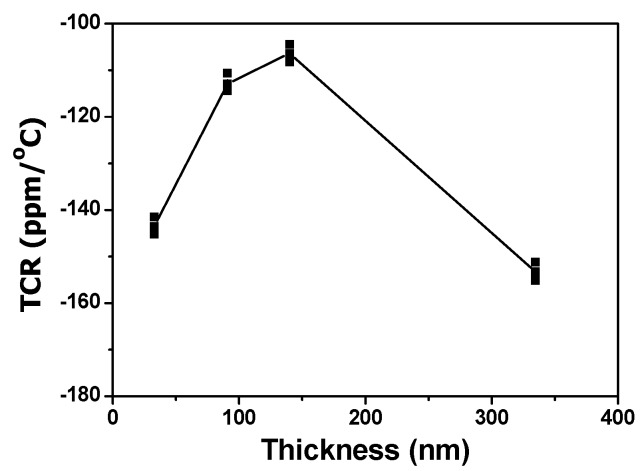
Effect of thickness on the variations of temperature coefficient of resistance (TCR) for the deposited NiCrSi thin-film resistors.

The TCR values of deposited NiCrSi thin-film resistors are shown in Figure 5 as a function of film’s thickness, using the measured results shown in Figure 4. All of the TCR values of deposited NiCrSi thin-film resistors has a negative number, meaning that resistance decreases with increasing measured temperature. For pure metals, this coefficient is a positive number, meaning that resistance increases with increasing measured temperature. The TCR value of Ni metal is 0.00017 and the TCR value of Cr metal is 13 × 10^−8^, respectively. Dhere *et al.* found that Ni–Cr thin films of low positive TCR value (less than 100 ppm/°C) were obtained at all thicknesses studied when the sum of the total atomic contents of chromium, oxygen and carbon reached 50%–55% [16]. For the elements silicon in single crystalline type, the TCR value is a negative number of about −0.04 (depending strongly on the presence of impurities in the material). Because of this, we believe the negative TCR value of NiCrSi thin-film resistors is caused by the addition of Si in Ni–Cr alloy, and Si can be added in the NiCr composition for shifting the TCR values to close 0 ppm/°C. The TCR shown in Figure 5 first shifted to small negative value as the thin films’ thickness increased from 30.8 nm to 140.1 nm and then shifted to large negative value as the thin films’ thickness increased to 334.7 nm. The reason for this result is not really known, the formations of unknown alloy or compound during the annealing process is the possible reason for this result.

Lacy’s proposal shows that as the thickness of thin-film resistors is thinner than 20 nm, the resistivity of thin-film resistors will exponentially decrease. As the thickness of thin-film resistors is thicker than 20 nm, the resistivity of thin-film resistors will linearly decrease. In a thin film material, if, as proposed, the thin films have smooth or even surfaces, the surface scattering (rather than other scatterings) is believed to be the main reason that will affect the resistivity of thin-film materials. In this study, the thickness of deposited thin films is thicker than 30 nm. We believe the thin films are thick enough and the bulk mean free path of the electrons in NiCrSi thin-film resistors is less than *t*/2. Therefore, only the partial electrons located at in the *t*/2 region of upper face and down face will be scattered by the thin films’ surfaces, and only partial electrons located at *t*/2 the mean free path of the average conduction electrons will be altered or the electrons will be scattered by the surface. The ratio of conduction electrons will be altered or be scattered decreased with increasing the thickness of NiCrSi thin films. We believe this is the reason that the resistivity of NiCrSi thin-film resistors linearly decreases with increasing thickness.

**Figure 6:**
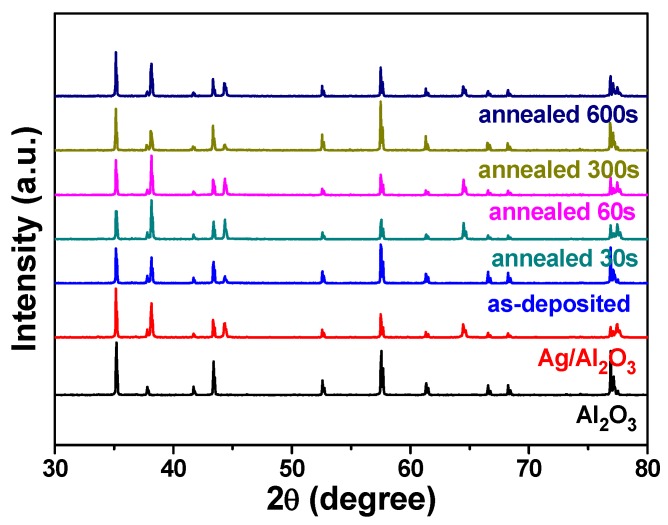
XRD patterns of annealed NiCrSi thin-film resistors, the deposition time was 60 min.

Figure 6 shows the XRD patterns of 60 min-deposited NiCrSi thin films as a function of annealing time. As Figure 6 shows, only the Ag and Al_2_O_3_ phases were observed and no other crystalline phases were observed. Because the NiCrSi thin films are annealed in N_2_ atmosphere, we believe that the oxidation will not happen during the annealing process. Those results suggest the annealing process has no effect on the crystallization of NiCrSi thin films but it will have the chance to densify the NiCrSi thin films.

**Figure 7:**
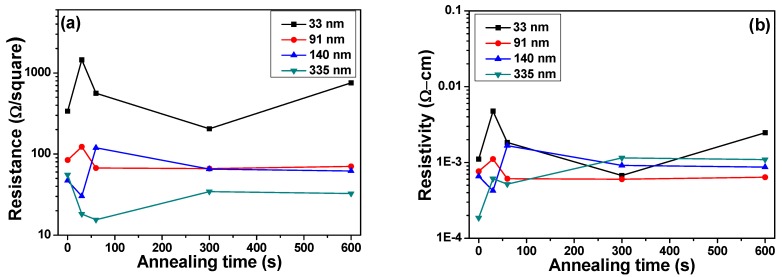
Variations of (**a**) resistance and (**b**) resistivity of 25 °C-measured NiCrSi thin-film resistors as a function of annealing time, respectively.

**Figure 8:**
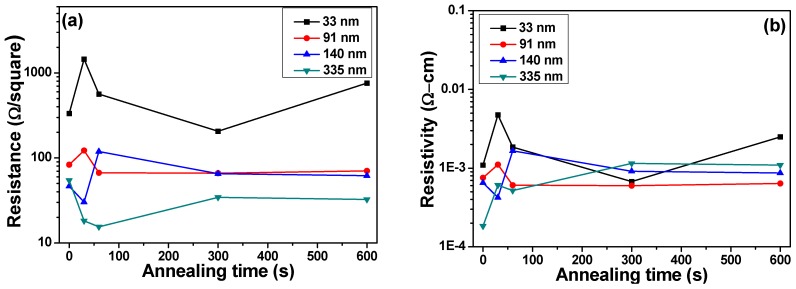
Variations of (**a**) resistance and (**b**) resistivity of 125 °C-measured NiCrSi thin-film resistors as a function of annealing time, respectively.

The variations of resistance and resistivity of NiCrSi thin-film resistors after annealing process are shown in Figure 7 for the deposited thin films measured at 25 °C and in Figure 8 for deposited thin films measured at 125 °C, respectively. Figure 7 and Figure 8 show that as the thickness of NiCrSi thin-film resistors was equal and more than 91 nm and as the annealing time was equal and more than 60 s, the resistance and resistivity would reach a stable value, independent of deposition time. Nocerinot and Singer find that the variations of resistance values were caused by two effects [17]. The first effect was adsorption of residual O_2_ gas during the deposition process. Because we deposited the thin films in the pure argon atmosphere, the effect of residual O_2_ gas on the properties of NiCrSi thin films can be neglected. The second effect was annealing of thin films, which would change the crystalline structure of thin-film materials or form chemical compound with thin-film materials. Thus, we anneal the NiCrSi thin films in the pure N_2_ atmosphere, the effect of chemical compound formed can also be neglected. However, the annealing process can stabilize the crystalline structure of deposition on thin films, because the resistivity and resistance of NiCrSi thin-film resistors will have a stable property after a period of annealing process.

**Figure 9:**
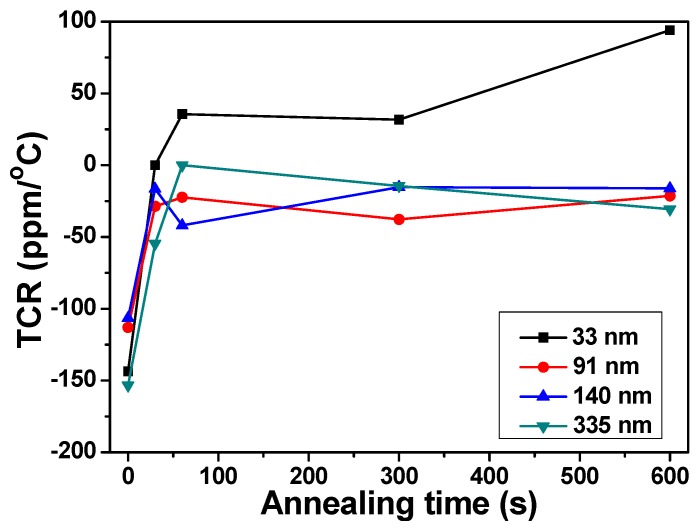
Variations of temperature coefficient of resistance of deposited NiCrSi thin-film resistors as a function of annealing time.

For applications of thin-film resistors, the temperature coefficient of resistance is hoped to be very close to zero, meaning that the resistance hardly changes at all with variations in temperature. In the past, the TCR values could be kept near zero for a wide variety of hybrid substrate materials with varied surface finishes by changing dopant concentration in the sputtering target. During the deposition process, the resistance and TCR value of thin-film resistors are also found to vary with both deposition and annealing parameters. Variations of TCR values of deposited NiCrSi thin-film resistors are shown in Figure 9 as a function of annealing time. Figure 9 shows that, as the annealing time was increased from 0 s to 60 s, the TCR value of the 33 nm-NiCrSi thin-film resistors changed from negative to positive; as the annealing time was increased from 60 s to 600 s, the TCR value changed from +31.7 ppm/°C to 94.2 ppm/°C. As the thicknesses of NiCrSi thin-film resistors were 91 nm, 140 nm, and 330 nm and as the annealing time was increased from 0 s to 60 s, the TCR values changed from −106.4~−153.3 ppm/°C to −41.9~0 ppm/°C; as the annealing time was increased from 60 s to 600 s, the TCR values were stable in the range of −14.5 ppm/°C to −37.7 ppm/°C. Nocerinot and Singer found that the effects of introducing oxygen into the Ni–Cr thin films during deposition process would decrease the TCR value [17]. However, we deposited and annealed the NiCrSi thin-film resistors in Ar and N_2_ atmosphere, respectively, the substrate used in this study is the high resistivity Al_2_O_3_, which has the resistivity higher than 10^8^ Ω-cm. Therefore, the effect of substrates will be neglected. Bayne found that the TCR value of standard 60/40 Ni–Cr thin-film resistors was around ~100 ppm/°C for constant sheet resistance films [18]. Those results suggest that before the annealing process the Si will dominate the characteristic of TCR, and after annealing process the effect of Ni–Cr Si will affect the characteristic of TCR, because the TCR value of NiCrSi thin-film resistors changes from negative to positive or shifts to near 0 ppm/°C.

## 4. Conclusions

As the deposition time was 15 min, 30 min, 60 min, and 150 min, the thickness of NiCrSi thin films was around 30.8 nm, 90.7 nm, 140.1 nm, and 334.7 nm, respectively. All of the NiCrSi thin films showed amorphous phase even the deposition time was 150 min, and even thin films annealed at 400 °C for 300 s NiCrSi also showed amorphous phase. The resistivity of deposited NiCrSi thin-film resistors first increased with thickness and reached a saturation value as the thickness was equal and more than 140.1 nm, and the resistivity linearly decreased with increasing NiCrSi thin-films’ thickness. When the annealing process was used, as the thickness of NiCrSi thin-film resistors was equal and more than 91 nm and as the annealing time was equal and more than 60 s, the resistance and resistivity would reach a stable value, independent of deposition time. As the thicknesses of NiCrSi thin-film resistors were 91 nm, 140 nm, and 330 nm, and the annealing time was more than 60 s, the TCR values were stable in the range of −14.5 ppm/°C to −37.7 ppm/°C.
